# Wild mink (*Neovison vison*) as sentinels in environmental monitoring

**DOI:** 10.1186/1751-0147-54-S1-S9

**Published:** 2012-02-24

**Authors:** Sara Persson, Björn Brunström, Britt-Marie Bäcklin, Hans Kindahl, Ulf Magnusson

**Affiliations:** 1 Div of Reproduction, Swedish University of Agricultural Sciences, P.O. Box 7054, SE-750 07 Uppsala, Sweden; 2 Department of Environmental Toxicology, Uppsala University, Norbyvägen 18 A, Uppsala, Sweden; 3 Dept of Contaminant Research, Swedish Museum of Natural History, P.O. Box 50007, SE-104 05 Stockholm, Sweden

## Abstract

**Summary:**

For monitoring of endocrine disruptors and other contaminants in terms of exposure levels and possible effects on the reproductive system, suitable sentinel species have to be used. The wild mink is putatively a good sentinel, as it is a semi-aquatic top predator present in all parts of Sweden and it is hunted extensively. When studying the wild mink from an environmental health perspective, one has to consider advantages and disadvantages with different ways of sampling. Field studies often generate large data sets and the variables measured may show large variation due to intrinsic and extrinsic biological factors; this must be adjusted for when analyzing data in order to increase the chances of identifying associations between exposure and health effects. With focus on our own data from studies in wild mink in Sweden, this article describes how the mink can be used in large scale screening of exposure and effects of contaminants.

## Background

Sentinel wildlife species can serve as indicators of environmental health and may provide information on trends in concentrations of pollutants and give early warnings about potential health hazards [1]. In addition to being a tool for assessment of exposure to and/or effects of environmental contaminants in wildlife, sentinels can indicate human health risks [2,3]. The selection of sentinel species is crucial for optimizing sensitivity and coverage in monitoring of chemical exposure and effects.

## The mink as a sentinel species

The mink was used in exposure and effect studies already in the 60’s and 70’s, when contaminant levels in wild mink were investigated and compared with those causing adverse effects in toxicological studies on farmed mink [4]. Effects in mink that were fed fish from the Miramichi River [5] and Great Lakes [6,7] were also studied. Through the 80’s, 90’s and 00’s, the amount of literature on toxic effects by chemicals in farmed mink and on the exposure of wild mink to pollutants has continuously expanded, as well as the literature on exposure of pollutants to wild mink. With the aim to expand upon the literature available upon the toxic effects, the role of the wild mink as a sentinel species in environmental health monitoring has recently been reviewed [8]. As many contaminants affect reproduction, the extensive amount of literature on mink biology and reproduction [9] favors the mink as a sentinel species.

The mink is a top predator, feeding mainly on fish, crayfish, rodents, frogs and birds such as waterfowl [10]. The habitat of the mink is relatively small, on average 2.5 km along a shore-line [11]. The semi-aquatic feeding habits in combination with the restricted habitat suggest that contaminant concentrations in a specimen well reflect the local contamination with many persistent pollutants.

In Sweden, the mink is an invasive species that is present in most parts of the country. An estimated amount of 11 500 mink was hunted in Sweden in 2007/2008 [12]. Swedish mink are most often hunted as a protective measure, rather than being trapped for their fur. Local hunting campaigns are sometimes initiated, as the mink can do substantial damage to populations of birds [13,14], mammals [15] and frogs [16]. As a result of the alien status of the mink, the legislation permits hunting all year round in unlimited numbers [17], which facilitates sampling.

## Practical aspects of using the mink as a sentinel species

Basu et al (2007) [8] recommend sampling of live animals, as repeated measurements and radiotelemetry are possible. Such study designs may be beneficial to meet the objectives of some studies, but are not suitable for large scale screening. In Sweden, live traps have to be attended two times per day, morning and evening [18], which is demanding in terms of labour; the number of collected samples would be quite limited because of the economic cost. Naturally, only a limited geographical area would be covered.

In order to sample a large amount of carcasses from large geographical areas, or from distant areas, it is advisable to turn to local hunters. In our experience, trappers are interested and prone to participate in sampling of mink. In Sweden, it is most common to use lethal traps or to hunt by dog and shotgun. The relatively small body of the mink, males weighing approximately 1100 g and females 620 g [19], makes the carcasses easy to handle and transport. In the sampling we are performing, we have chosen to work with frozen carcasses. The reason for this is that it is considerably easier for the hunter to save mink carcasses continuously and send all mink at the same time. Keeping the carcasses frozen also facilitates planning of work for the person doing the necropsies. To sample fresh carcasses would demand a lot more work from the hunter and would most probably result in many carcasses being more or less rotten, as transportation often takes at least a day or two. It would also demand that the person doing the necropsies is at constant standby. Collecting frozen carcasses has been successful in our project as we have sampled over 500 mink during the past five years. The major drawback of freezing the animals is that it limits the possibilities of doing sophisticated histology, as the tissue suffers from artefacts due to disruption of cell membranes by freezing and thawing. Even so, we have been successful in measuring the diameter of the seminoferous tubuli in many of the mink testicles [20]. We were also able to evaluate sperm morphology on samples taken from the epididymides. The sperm had damaged acrosomes because of freezing, but besides this it was possible to record all common abnormalities.

## Variation in data from environmental monitoring

Data from field studies often show a lot of variation in both biological variables and body burden of contaminants. This large variation is influenced by intrinsic and extrinsic factors. Hence, it is critical to identify these factors and to adjust for them when analyzing data. This will increase the precision of exposure and effect data and will facilitate identification of subgroups within populations that are at the highest risk of adverse health effects due to chemical exposure.

Variations in concentrations and patterns of contaminants in wildlife are not only due to temporal and spatial variation, but also biological factors such as for example reproductive stage, age, sex, habitat use, migration and diet [21]. Metabolism is also an important factor, as it includes events such as growth, hibernation and lactation. All these biological and physiological factors may correlate with each other. For example, seasonal variation in contaminant concentrations in a species may be contributed to metabolism (for example lipids), diet, migration and reproduction. Variation in physiological data from field studies could be attributed to differences in reproductive stage, age, sex, body condition etc. Variation could also derive from pathology, due to xenobiotics, infections etc.

## Own studies using the wild mink as a sentinel

Our sampling design results in collection of both male and female mink from different areas and of different ages all year round. Multiple regression analysis was chosen to characterize which factors that influence physiological traits and exposure levels. In our first study, season and age had significant influence on many of the selected reproductive traits in male mink, but no significant effect from nutritional status was found [20]. Data on brominated flame retardants (BDEs) showed that among many factors studied, nutritional status was a factor that significantly influenced the concentration (p=0.0015) (In manuscript). By including these influencing factors into a multiple regression model, preliminary data showed a significant positive correlation (p=0.02) between ∑BDE concentration and testicle weight [22].

## Conclusions

The mink is a good sentinel species, both based on its biological characteristics and for practical reasons. With the aim to sample large numbers of mink over time, from both large and small geographical areas, we successfully used local hunters and handled frozen carcasses. Factors such as age, season and nutritional status must be taken into account when monitoring effects on the reproductive system in the wild mink.

## Competing interests

The authors declare that they have no competing interests.

## Authors' contributions

Please see sample text in the instructions for authors.

SP wrote most of the manuscript. BB, BMB and HK commented on the manuscript. UM planned the manuscript together with SP and contributed to writing the manuscript. All authors read and approved the final manuscript.
